# Influence of freezing and heating conditions on grape seed flavan-3-ol extractability, oxidation, and galloylation pattern

**DOI:** 10.1038/s41598-022-07925-7

**Published:** 2022-03-09

**Authors:** Joshua VanderWeide, Filippo Del Zozzo, Esmaeil Nasrollahiazar, James A. Kennedy, Enrico Peterlunger, Laura Rustioni, Paolo Sabbatini

**Affiliations:** 1 Wine Research Centre, Faculty of Land and Food Systems, The University of British Columbia, Vancouver, BC Canada; 2 Department of Horticulture, Plant and Soil Sciences Building, Michigan State University, East Lansing, MI 48824 USA; 3 Dipartimento di Scienze Agroalimentari, Ambientali e Animali, Università di Udine, via delle Scienze 206, 33100 Udine, Italy; 4 Function Phenolics LLC, PO Box 1443, Corvallis, OR 97339 USA; 5 Dipartimento di Scienze e Tecnologie Biologiche ed Ambientali, Centro Ecotekne, Università del Salento, via Provinciale Monteroni, 73100 Lecce, Italy

**Keywords:** Plant sciences, Secondary metabolism

## Abstract

In cool-climate viticulture, the short growing season can influence grape seed maturation by reducing the apparent oxidation of flavan-3-ol monomers and associated increase in seed browning. A reduction in seed maturation increases the potential extraction of flavan-3-ol monomers into wine during maceration operations, heightening bitterness. Here, we carried out a 2 × 2 factorial experiment to test the ability of freezing and heating treatments to advance maturation (decrease flavan-3-ol, improve browning) of (*Vitis vinifera* L.) Pinot noir and Cabernet Sauvignon seeds over a 24-h incubation period. Only freezing significantly increased seed browning in both cultivars. Subsequent correlations with seed flavan-3-ol monomer concentrations suggest that freezing enhanced the oxidation of these compounds. Interestingly, natural ripening and freezing reduced galloylated flavan-3-ol monomers to a greater extent than non-galloylated ones. This study provides new information regarding the susceptibility of flavan-3-ol monomers to freezing and heating, and also suggests that freezing can advance the maturation the seeds of under-ripe red *vinifera* grapes.

## Introduction

During grape ripening, seeds shift in color from yellow to dark brown. The change in color has been associated with the oxidation of phenolic compounds, which are located in the outer integument cell layer^1^. The seed color change is also accompanied by seed water loss and a reduction in seed weight^2,3^. Grape seed phenolics are predominately represented by flavan-3-ols^4^. The compounds most susceptible to oxidation are those having the *ortho*-dihydroxy substitution pattern, particularly those in large concentrations such as ( +)-catechin, (−)-epicatechin, and epicatechin-3-*O*-gallate^5,6^. Oxidative polymerization leads to an apparent decrease in the mean degree of polymerization (mDP) of seed tannins during berry ripening^7^, which decreases their extractability in wine^8,9^.

The influence of grape seed tannins on wine quality has been investigated thoroughly through studies that either add or remove grape seeds or seed-derived tannins to wine. The presence of seed tannins improved the color and pigmented polymer stability of Cabernet Sauvignon wines compared to tannins from skins or without tannin fractions from either seeds or skins^10^. Likewise, seed removal in Monastrell wines decreased color intensity^11^. Canals et al. revealed the positive relationship between seed presence and wine anthocyanin content, and also showed a correlation with wine astringency and bitterness^12^. The total concentration of seed phenolics in wine was related to increased wine bitterness, but not astringency^13^, however, more recent work showed that cultivars having a greater proportion of seed weight to berry weight produce more astringent wines^14^. Additionally, high concentrations of seed phenolics mask desirable wine aromas^11,15,16^. This may be why Sáenz-Navajas et al. revealed that the presence of astringency-related phenolics, including procyanidins, correlated negatively with consumer preference of red wine^17^. Together, this information suggests that while flavan-3-ols are important to red wine color and organoleptic properties, excessive concentrations can compromise wine flavour and quality.

In seasons with low growing degree day accumulation, cool climate-grown red *vinifera* cultivars can fail to reach harvest-quality maturity^18^. This results in low sugar concentrations and pH in the fruit, the latter of which exacerbates astringency perception in the final wine^19^. Additionally, seeds of underripe fruit contain higher and more extractable seed flavan-3-ol monomers, as well as procyanidin concentrations with a greater mDP^14,20–22^, which can negatively impact wine quality due to increased astringency and bitterness^23–25^. In cool climate viticulture, grapevines are manipulated using management practices to advance ripening and improve harvest fruit quality^26–29^. However, these approaches have little effect on seed maturity^30–32^, which presents a unique challenge for winemakers. This suggests the need for a technique to alter bitter and astringent flavan-3-ols compounds to improve red wines made with underripe fruit. One such enological approach is to remove seeds from red wine ferments that sink to the bottom of fermentation tanks to limit extraction of seed flavan-3-ols (i.e.: early seed removal). Lee et al. reported a slight decrease in terminal galloylated catechins using this approach, and a subsequent increase in mDP; however, little difference in wine chemistry and quality were observed^33^. Based on these results, it was hypothesized that flavan-3-ols from seeds are thoroughly extracted from seeds prior to settling^33,34^. Likewise, other enological approaches to improve tannin quality in red wine have fallen short of addressing this issue^35^.

Our previous work reported that a “freeze–thaw” treatment advanced the maturation of grape seeds by browning the seed coat of ten red *Vitis vinifera* L. cultivars^36^. We hypothesized that this was due to enhanced oxidation of phenolic compounds. In a second study, we observed that the change in seed color accompanied a significant alteration in phenolic compounds. This suggested that ethanol extractable phenolics likely present in the vacuole were significantly reduced^37^. This was confirmed by microscopy images, which revealed dark masses near cells with compromised structure; indicative of phenolic oxidation^37^. In the current study, we expanded the experimental design and evaluated the seed extract phenolics to understand how concentrations of individual flavan-3-ols respond to “freezing,” “heating” (thawing temperature), and “incubation time” (thawing time). Our hypothesis was that, in addition to freezing, the higher thawing temperature and greater thawing time would enhance flavan-3-ol oxidation and advance the maturation of both Pinot noir and Cabernet Sauvignon seeds.

## Results and discussion

### A. Seed color is significantly browned by treatment conditions

A color index (CI) was calculated to link all the three colorimeter parameters and better visualize the change in seed color during natural ripening and in response to treatments. Natural ripening led to a significant decrease in individual colorimeter parameters as well as in seed browning (lower index) in both PN and CS (Supplemental Figs. 1–4). However, the change in CI was over 7-times greater in CS than PN (Table 1). This might be linked to higher concentrations of flavan-3-ols in PN seeds compared to other *vinifera* cultivars^39^. This result may also reflect the larger size of PN seeds compared to other *vinifera* cultivars, and therefore, a difference between PN and CS in the extractability of flavan-3-ols and their susceptibility to oxidation^4^. Finally, PN berries were slightly less ripe (−1.5°Brix) than CS despite harvesting fruit based on identical maturity in the field, which could influence the impact of natural ripening (Supplemental Table 1).

**Table 1:** Impact of natural ripening, incubation time, freezing treatment, and heating treatment on the color index of Pinot noir and Cabernet Sauvignon seeds.

Treatment^a^	Pinot noir				Cabernet Sauvignon			
Natural ripening	Veraison	Harvest	%change	p-value	Veraison	Harvest	%change	p-value
	123	120	−2.44	**0.022**	123	99.5	−19.1	** < 0.001**

Incubation	0 min	3 h	%change	p-value	0 min	3 h	%change	p-value
	122	115	−5.74	** < 0.001**	110	103	−6.36	**0.010**

Incubation	0 min	6 h	%change	p-value	0 min	6 h	%change	p-value
	122	104	−14.8	** < 0.001**	110	103	−6.36	**0.004**

Incubation	0 min	24 h	%change	p-value	0 min	24 h	%change	p-value
	122	102	−16.4	** < 0.001**	110	100	−9.09	** < 0.001**

Freezing	20 °C	−20 °C	%change	p-value	20 °C	−20 °C	%change	p-value
	114	105	−7.89	** < 0.001**	109	102	−6.42	** < 0.001**

Heating	20 °C	40 °C	%change	p-value	20 °C	40 °C	%change	p-value
	111	108	−2.70	0.069	105	105	0	0.986

Significant values are in bold.

^a^Data from untreated seeds was used for analysis of natural ripening, while data from all treatments was used for analysis of other parameters.

**Figure 1:**
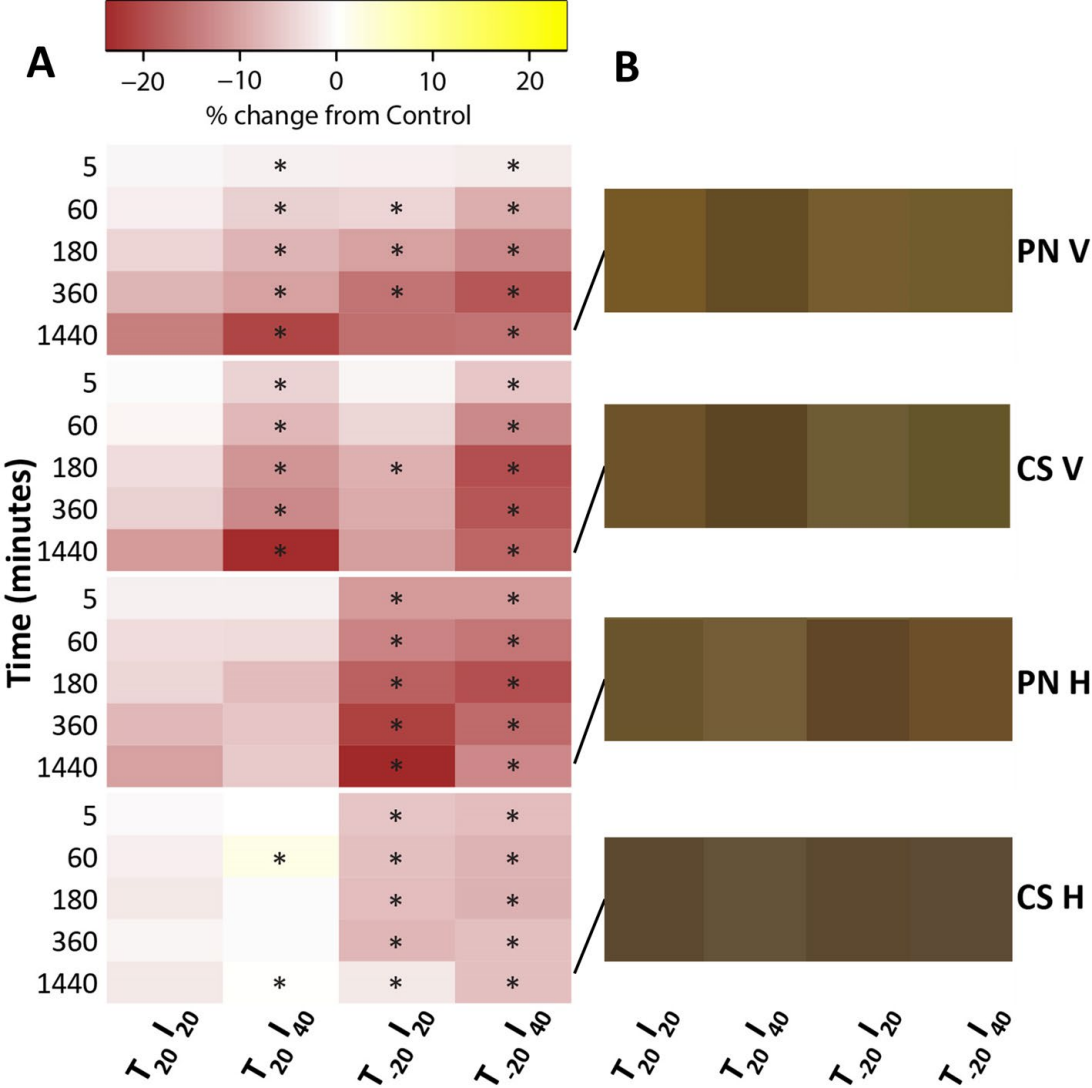
**(A)** Heatmap revealing the percent change of the seed color index over 24 h in response to freezing and heating treatments. **(B)** Images representing actual seed color from colorimeter (L*, C*, h*) values after 24 h of treatment exposure. The treatment effect was analyzed by one-way ANOVA, and when the differences were significant, means were separated with Tukey’s HSD test (*p* < 0.05). Treatments changed significantly from the control (T_20_I_20_) are marked with an asterisk, *, p < 0.05; **, p < 0.01; ***, p < 0.001. T_20_I_20_, no freezing and thawing at 20 °C; T_20_I_40_, no freezing and thawing at 40 °C; T−_20_I_20_, freezing at −20 °C and thawing at 20 °C; T−_20_I_40_, freezing at −20 °C and thawing at 40 °C; sampling time; *T* freezing temperature; *I* incubation temperature. *PN V* Pinot noir at veraison; *CS V* Cabernet Sauvignon at veraison; *PN H* Pinot noir at harvest; *CS H* Cabernet Sauvignon at harvest.

The incubation period impacted seed color for both cultivars significantly (Table 1, Fig. 1). However, for each cultivar and sampling time, the point at which treatments changed from ‘time 0’ was different. Seeds from all treatments in both cultivars browned significantly after 3 h at veraison (Supplemental Fig. 4). Meanwhile at harvest, seeds required 24 h and 6 h for PN and CS, respectively. For this reason, there was a significant effect of the incubation time on seed color in both cultivars (Table 1). Similar browning between cultivars during seed incubation (without the presence of pulp and skin tissues) suggests that the differences observed during natural ripening between PN and CS were attributed to physical structure of the seeds.

In both PN and CS, freezing treatments induced an immediate browning of the seed color that lasted until 6 h (Fig. 1, Supplemental Figs. 1–4). While effects were observed at veraison, both freezing treatments had a more significant impact on seeds at harvest. This confirms our previous work which reported a significant CI change after 3 h post-freezing^36,37^. The cause for this greater effect on seeds at harvest is intriguing, as they are more lignified than at veraison, and lignification reduces the susceptibility of plant cells to freezing damage^40^.

The effect of heating was impactful at veraison, with T_20_I_40_ having the darkest seeds in both cultivars after 24 h (Table 1, Fig. 1). In contrast, harvest samples were unaffected by heating, contributing to the non-significant effect of heating on color when both timings are considered (Table 1). As seeds ripen, the inner-most cell layers of the outer integument lignify, making the seed less penetrable^1^. This process diminishes the conductance of heat through these cell layers, as is important during seed dormancy^40^.

### B. Natural ripening and freezing treatment differentially influence the seed flavan-3-ol profile by the gallyolation pattern of compounds

**Figure 2:**
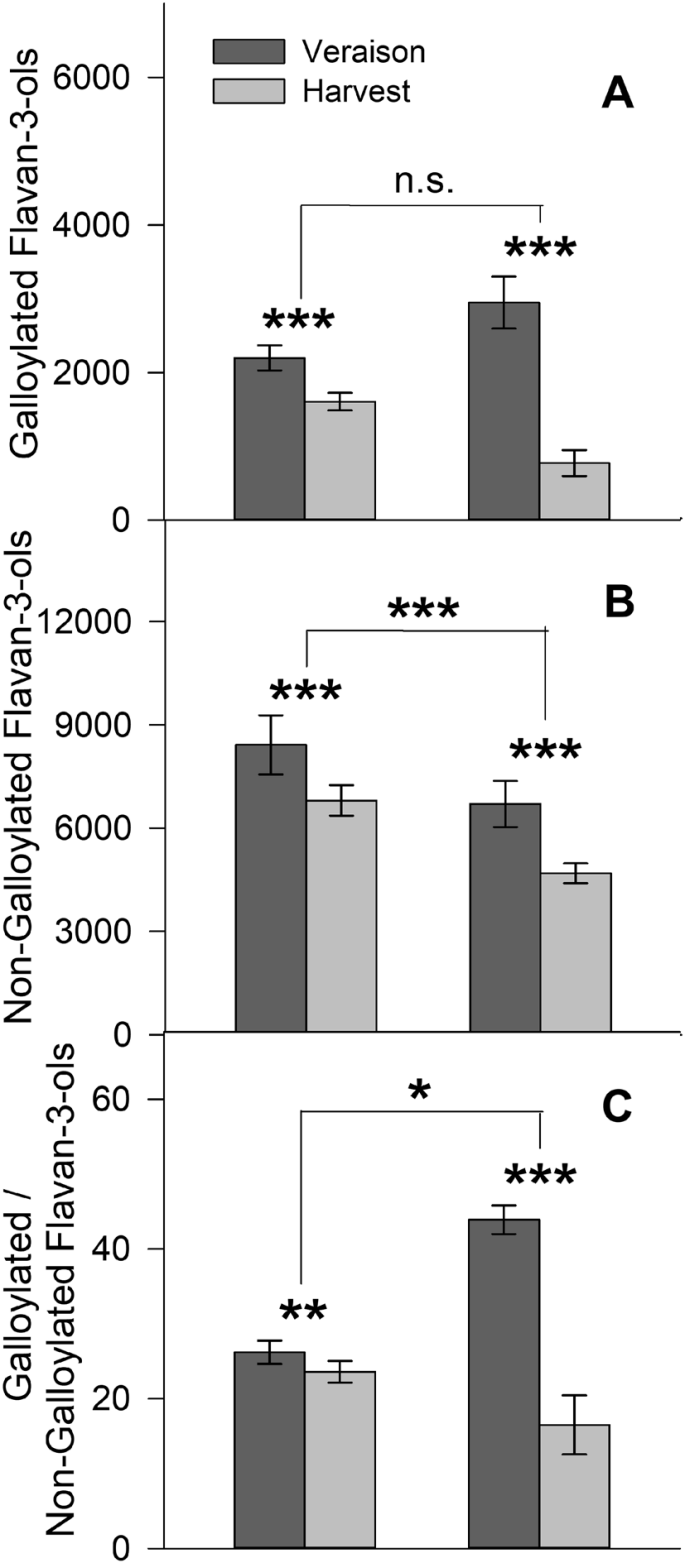
Influence of cultivar (Pinot noir, left; Cabernet Sauvignon, right) and natural ripening on the concentration (µg/g seeds FW) of **(A)** galloylated flavan-3-ols, **(B)** non-galloylated flavan-3-ols, and **(C)** the ratio between galloylated and non-galloylated flavan-3-ols in seed extracts. Data from untreated seeds was used for analysis. Data is expressed as mean standard ± deviation. Treatments changed significantly from the control (T_20_I_20_) are marked with an asterisk, *, p < 0.05; **, p < 0.01; ***, p < 0.001.

Among variables in this experiment, cultivar accounted for the largest variability in the seed extract flavan-3-ol profile (Table 2). The majority of compounds influenced by cultivar were non-galloylated, while galloylated flavan-3-ols explained little variance (Table 2, Fig. 2A,B). This may be because non-galloylated flavan-3-ols, such as ( +)-catechin and (−)-epicatechin, are represented in the highest concentrations in grape seeds and vary greatly between cultivars^4^. Likewise, Núñez et al. reported that non-galloylated flavan-3-ols are negatively correlated with mDP (differ greatly by berry maturity)^41^. This suggests that the substantial oxidation and polymerization experienced by these compounds during fruit ripening may also influence their cultivar-dependent concentrations^2^. Meanwhile, alterations in mono-galloylated flavan-3-ols are thought to be more season-dependent^41^.

**Table 2:** Variance in the seed flavan-3-ol profiles of Pinot noir and Cabernet Sauvignon seed extracts according to treatments and their interactions.

Flavan-3-ol compound	Cultivar (C)	Natural Ripening (NR)	Incubation Time (IT)	Freezing (F)	Heating (H)	C x NR	C x IT	C x F	C x H	NR x T	NR x F	NR x H	IT x F	IT x H	F x H	3-way interactions (sum)	4-way interactions (sum)	5-way interactions (sum)	Unexplained Variance	Total
Column average	38.3	23.8	1.12	2.29	0.780	22.1	0.450	0.410	0.450	0.110	2.27	0.040	0.400	0.260	0.280	5.32	1.21	0.160	0.210	100
Procyanidin C	75.3	4.16	0.020	0.020	0.040	14.0	0.170	0.230	0.120	0.020	0.900	0.000	0.070	0.190	0.260	3.95	0.480	0.030	0.100	100
Procyanidin B2	65.7	12.6	0.100	0.140	0.250	15.5	0.110	0.00	0.00	0.080	0.490	0.000	0.050	0.220	0.020	3.55	1.03	0.010	0.140	100
(−)-epicatechin	62.6	12.0	0.230	0.320	0.150	7.66	0.240	0.060	0.730	0.170	2.20	0.160	0.010	0.670	0.390	10.7	1.36	0.160	0.230	100
Procyanidin A	54.8	25.1	0.770	0.00	0.400	9.76	0.260	0.00	0.210	0.180	1.17	0.000	0.210	0.100	0.010	5.69	1.14	0.050	0.180	100
( +)-catechin	44.7	27.6	0.760	0.150	4.16	0.710	1.78	0.700	2.08	0.020	0.970	0.060	1.36	0.580	0.550	10.1	3.34	0.190	0.220	100
Epicatechin gallate	2.76	48.9	1.92	9.22	0.280	33.9	0.070	0.010	0.030	0.070	0.680	0.030	0.090	0.110	0.010	1.21	0.57	0.030	0.060	100
Procyanidin C2 gallate	0.510	25.8	4.83	7.60	0.930	34.7	0.720	2.31	0.400	0.250	11.0	0.000	1.38	0.120	0.870	5.75	1.43	0.740	0.700	100
Procyanidin B2 3’-*O*-gallate	0.250	34.4	0.330	0.830	0.030	60.7	0.250	0.00	0.010	0.070	0.730	0.040	0.050	0.110	0.100	1.62	0.360	0.080	0.060	100

In general, natural ripening and freezing treatments induced a larger relative change to galloylated compounds compared to non-galloylated ones (Fig. 2, Supplementary Tables 2, 3). With respect to natural ripening, the compound with the greatest relative decrease was epicatechin-3-*O*-gallate, a result observed previously^7,42^. Additionally, there was a less visible effect on non-galloylated compounds. This led to both parameters having a significant impact on the ratio of galloylated to non-galloylated flavan-3-ols (Table 3). Galloylated flavan-3-ols are formed by a condensation reaction using gallic acid^43^. This structure provides three additional hydroxyl groups capable of donating electrons. Galloylated flavan-3-ols have a higher free radical quenching capacity (DPPH) compared to non-galloylated ones, such as catechin and epicatechin^44,45^. However, the oxygen radical absorbance capacity (ORAC) assay revealed the opposite trend^45,46^. This difference in antioxidant results may lie in the enhanced ability of galloylated flavan-3-ols to penetrate lipid bilayers due to their more lipophilic nature^25,47^. Our previous work showed that freezing disrupts cell membranes and walls^37^, suggesting that this may allow galloylated compounds to become exposed to oxygen. Additionally, flavan-3-ols with galloyl groups were less stable at neutral pH^25^. More recently, Kim et al. revealed that phenolics with a pyrogallol group were highly oxidizable under a pH  10^48^. This suggests that seeds out of wine-like (low pH) solution may promote oxidation of galloylated phenolics.

**Table 3:** Impact of natural ripening, freezing treatment, and heating treatment on the ratio of galloylated to non-galloylated flavan-3-ols in Pinot noir and Cabernet Sauvignon seed extracts.

Treatment^a^	Pinot noir				Cabernet Sauvignon			
Natural ripening	Veraison	Harvest	%change	p-value	Veraison	Harvest	%change	p-value
	26.2	23.6	−9.92	**0.002**^b^	43.9	16.5	−62.4	** < 0.001**

Freezing	20 °C	−20 °C	%change	p-value	20 °C	−20 °C	%change	p-value
	24.9	19.7	−20.9	** < 0.001**	29.6	21.7	−26.7	**0.011**

Heating	20 °C	40 °C	%change	p-value	20 °C	40 °C	%change	p-value
	22.6	22.0	−2.65	0.504	26.2	25.0	−4.58	0.715

^a^Data from untreated seeds was used for analysis of natural ripening, while data from all treatments was used for analysis of other parameters.

Significant values are in bold.

Heating treatment had an inconsistent effect on individual flavan-3-ol compounds. B-type, C-type procyanidins, and total procyanidins were significantly increased and decreased in PN and CS, respectively (Supplementary Table 4). This led to no effect on the ratio of heating on galloylated to non-galloylated flavan-3-ols extracted from seeds in either cultivar (Table 3).

### C. Relationships between seed color and flavan-3-ols composition

The Pearson’s correlation coefficients between the seed color index and seed flavan-3-ol groups (galloylated, non-galloylated, and their ratio) are displayed in Table 4. When the data from both cultivars were considered together, all flavan-3-ol groups were correlated strongly with the color index (CI) (Table 4). This is reflective of the relationship between seed flavan-3-ol concentration and color^6^. However, when examined on a cultivar-dependent basis, these correlations were only completely upheld in CS. In PN, the relationship between the CI and total flavan-3-ols, galloylated flavan-3-ols, and the ratio of galloylated flavan-3-ols to non-galloylated compounds were significant. But there was no relationship between CI and non-galloylated flavan-3-ols. This suggests that despite the large decrease in non-galloylated flavan-3-ols during natural ripening, that galloylated flavan-3-ols may play a large role in seed color determination in proportion to their concentration. Given the location of compounds largely comprising the non-galloylated fraction in vacuoles of cells, it may be that differences in extractability of compounds may be influencing this difference between cultivars^4^. Future works will seek to establish whether the structural characteristics of seeds influence seed coloration during berry ripening.

**Table 4:** Pearson correlations between the color index of Pinot noir and Cabernet Sauvignon seeds and the concentrations of flavan-3-ol groups in seed extracts.

Phenolic group	Both cultivars R^2^	*p*-value	Pinot noir R^2^	*p*-value	Cabernet Sauvignon R^2^	*p*-value
Total^a^	0.603	** < 0.001**	0.289	**0.014**	0.776	** < 0.001**
Galloylated	0.663	** < 0.001**	0.417	** < 0.001**	0.792	** < 0.001**
Non-galloylated	0.510	** < 0.001**	0.208	0.079	0.740	** < 0.001**
Galloylated/non-galloylated	0.560	** < 0.001**	0.368	**0.001**	0.755	** < 0.001**

^a^All data was utilized for correlation analysis.

Significant values are in bold.

### D. Implications of treatments on grape and wine quality

Grape growers often utilize seed color and organoleptic properties as a basis for harvest maturity of fruits. This study reveals that seed color may not a be a sufficient marker alone to determine grape ripeness due to the large variability among cultivars in the concentration of compounds related to both of these parameters^6^. It is well established that galloylated flavan-3-ols are a source of astringency in red grapes wines^14,25,49^. Flavan-3-ol monomers are also well known to impact bitterness in wine^17^. The results from this study provide some implications for wine quality based on these facts. Wines made with seeds subjected to freezing treatment should be less bitter and astringent due to the significant decrease in galloylated phenolics, which are also understood to more strongly interact with bitter taste receptors due to their lipophilic nature^25^. However, freezing may in turn promote oxidative polymerization of procyanidins; a process known to enhance wine astringency^24^. The degree of procyanidin polymerization and galloylation were not analyzed in this study but will be a focus of future works. Likewise, given the potential differences in treatment effectiveness in must or wine solution^50^, future works will consider this aspect to truly understand whether this technique could positively influence red wine quality.

To conclude, this work characterized the response of seed flavan-3-ols to natural ripening and several treatments aimed to advance the maturation of seeds of Pinot noir and Cabernet Sauvignon. Seeds were separated from fruits and subjected to a 24-h time-course study following the subjugation of seeds to 2 × 2 factorial treatments of no freezing (20 °C) or freezing (−20 °C) prior to the time-course and no heating (20 °C) or heating (40 °C) during the time course. Cultivar had a large impact on the concentrations of non-galloylated flavan-3-ols, while natural ripening and freezing had a greater effect on galloylated flavan-3-ols than non-galloylated ones. Finally, heating induced negligible changes to compounds, but primarily non-galloylated ones. This study provides new information regarding the sensitivity of flavan-3-ols oxidation to freezing and heating, and suggests a novel approach to improve the quality of seed-derived flavan-3-ols in underripe red *vinifera* cultivars. Future works should evaluate the oenological potential of this technique.

## Materials and methods

### Experimental location and plant material

Based on our previous studies evaluating the impact of a “freeze–thaw” treatment on seed color and phenolics change^36,37^, two *Vitis vinifera* L. cultivars were chosen for the experiment; Pinot noir (PN) and Cabernet Sauvignon (CS), which display high and low seed flavan-3-ol monomer and procyanidin concentrations during maturation, respectively^4^. PN and CS fruit used for the experiment were collected in a commercial vineyard located in Southwest Michigan, 12 Corners Vineyards and Winery, Benton Harbor, MI, 49022, United States (lat: 42.08°, long: −86.22°, elevation: 227 m) during the 2019 growing season. At veraison (~ 11°Brix, 50% color change, 19 August) and harvest (~ 20°Brix, 27 September), 15 representative basal clusters were sampled for each cultivar, placed in plastic bags, and returned immediately to campus in coolers.

### Experimental design and seed preparation

The experiment was conducted according to a 2 × 2 factorial design (freezing, heating) with two cultivars (Pinot noir, Cabernet Sauvignon) and two sampling times (veraison, harvest). From the clusters collected at each sampling time, 1,200 berries were selected (for each cultivar) and distributed equally among 12 trays representing three replications of four treatments. The groups of 100 berries were manually adjusted to reach a similar weight and average berry color. Groups were then placed in separate Ziploc® bags and crushed. The seeds were separated from the pulp, rinsed with DI water, blotted dry with a KimWipe^®^, and immediately evaluated for color analysis (2.4). Meanwhile, juice was stored for later analysis of basic fruit quality components (2.3). Each group of 100 berries was randomly assigned to one of four treatments, with each treatment having three replications. The first treatment (T_20_I_20_), considered as the control, consisted of seeds subjected to “no freezing” (20 °C) and incubation at 20 °C. The second treatment (T_20_I_40_) was also not frozen (20 °C) but incubation at 40 °C. For the third treatment (T_-20_I_20_), seeds were frozen at − 20 °C (T_-20_) for 12 h and then incubated at 20 °C. The fourth treatment (T_-20_I_40_) included freezing seeds at − 20 °C for 12 h and incubation at 40 °C. Following seed removal and cleaning, seeds from each treatment were spread in a single layer across a 6 cm plastic weigh boat and incubated for 24 h. For I_40_ treatments, weigh boats containing seeds were kept in a laboratory convection oven at 40 °C and removed only for measurements during the time course study. For T_-20_ treatments, seeds in weight boats were placed in a − 20 °C freezer over-night (12 h) to ensure complete freezing of tissues. The following morning, weigh boats were removed from the freezer, and the time course study was initiated. The time course study consisted of taking seed colorimeter and chemical analysis samples at 0 min, 5 min, 1 h, 3 h, 6 h, and 24 h during the incubation time. At each point, seeds were weighed, mixed within the weigh boat, subjected to color and chemical analysis, and quickly returned to the incubation experimental treatments.

### Basic fruit analysis

Juice of PN and CS fruit previously placed in a 50 mL centrifuge tube was allowed to reach room temperature. Then, juice was centrifuged at 20 °C for 5 min at 1000×*g* prior to analysis. Total soluble solids and total acidity (tartaric acid equivalents) were analysed with an ATAGO digital refractometer specific to grape (ATAGO, Tokyo, Japan) and pH was measured with a 370 Thermo Orion pH meter (Thermo Fisher Scientific, Inc., Waltham, MA).

### Color analysis

Immediately after seed preparation, seeds were placed on a plastic weigh boat for incubation. Color measures occurred after 5 min, 1 h, 3 h, 6 h and 24 h during the incubation and after the freezing treatment. At each time point, a Konica Minolta Chroma Meter CR-400 (Konica Minolta, Osaka, Japan) was used to measure the seed color, and the weigh boat was not visible through the seeds. Afterward, seeds were mixed and re-settled into the weigh boat. This process was repeated an additional nine times, and the 10 technical replicates were averaged. These average values from each parameter (L, lightness; C, chroma; h, hue) for all samples were combined into a single color index parameter using factor analysis according to our previous work^37^. The following factor loading coefficients (α = 0.859, β = 0.795, γ = 0.731) were produced from the dataset.

### Flavan-3-ols extraction and analysis

After the analysis of seed color at each time point, a subsample of 20 seeds from each treatment at different time points during the incubation time were placed in a 50 mL centrifuge tube containing 20 mL of extract solution (80% v/v MeOH, 0.1% v/v formic acid) to extract seed flavan-3-ols. The covered tubes were wrapped with aluminum foil to prevent exposure to light and placed on a rotating platform at room temperature (20 °C) for 24 h. After 24 h, the extract solution was poured into another tube and stored at −20 °C, while 15 mL of new extraction solution was added to the seeds and rotated for three hours. After this time, the extract solutions were combined and stored at −20 °C. The extract solution was subjected to LC–MS using a Waters Acquity UHPLC interfaced to a Waters Xevo G2-XS Q-ToF mass spectrometer. Sample (10 μL) was injected onto a Waters HSS-T3 UHPLC column (2.1 × 100 mm, 1.7 μm particle size) held at 40 °C using a binary gradient of water with 0.1% v/v formic acid (solvent A) and acetonitrile (solvent B). The solvent flow rate was 0.3 mL/min; it was started at time 0 with 100% A, held for 0.5 min at 100% A, ramped to 50% B at 6 min, ramped to 99% B at 6.5 min, held at 99% B until 8.5 min, reverted back to the starting condition of 100% A at 8.51 min, and held until 10 min. Compounds were ionized by electrospray ionization in negative-ion mode with a capillary voltage of 3.0 kV, a cone voltage at 35 V, a source temperature of 100 °C, and a desolvation temperature of 350 °C. Data were acquired using a data-independent MSE method that consisted of two separate acquisition functions, one with no collision energy and the other with a collision-energy ramp of 20 − 80 V. Compounds were identified on the basis of their accurate masses, relative retention times, and fragmentation patterns. Peak areas were obtained using Quanlynx (part of the Waters Masslynx software). Standards of **( +)-**catechin and procyanidin B1 were used to quantify classes of flavan-3-ol monomers and procyanidins, respectively.

### Data elaboration and statistical analysis

The color index (CI) was determined according to our previous work^37^, and statistical analysis was conducted using SPSS statistical software (2020, version PASW Statistics 24, SPSS, Inc. Chicago, IL; https://www.ibm.com/products/spss-statistics-gradpack). The heatmaps were generated using RStudio version 3.6.2^38^ and data from treatments tested for significance from the control treatment using t-test (*p* = 0.05). Our experimental research and field studies on cultivated plants, complies with relevant institutional, national, and international guidelines and legislation.

## Supplementary Information

Supplementary Figures.

Supplementary Information.

## Data Availability

Data may be made available upon reasonable request.
