# Supplementary material for: Influence of freezing and heating conditions on grape seed flavan-3-ol extractability, oxidation, and galloylation pattern
Source: Sci Rep. 2022 Mar 9;12:3838. doi: 10.1038/s41598-022-07925-7 (PMC8907288; doi:10.1038/s41598-022-07925-7)
Supplement: Supplementary file 1 — Supplementary Figures. [file 41598_2022_7925_MOESM1_ESM.pptx]

## Slide 1
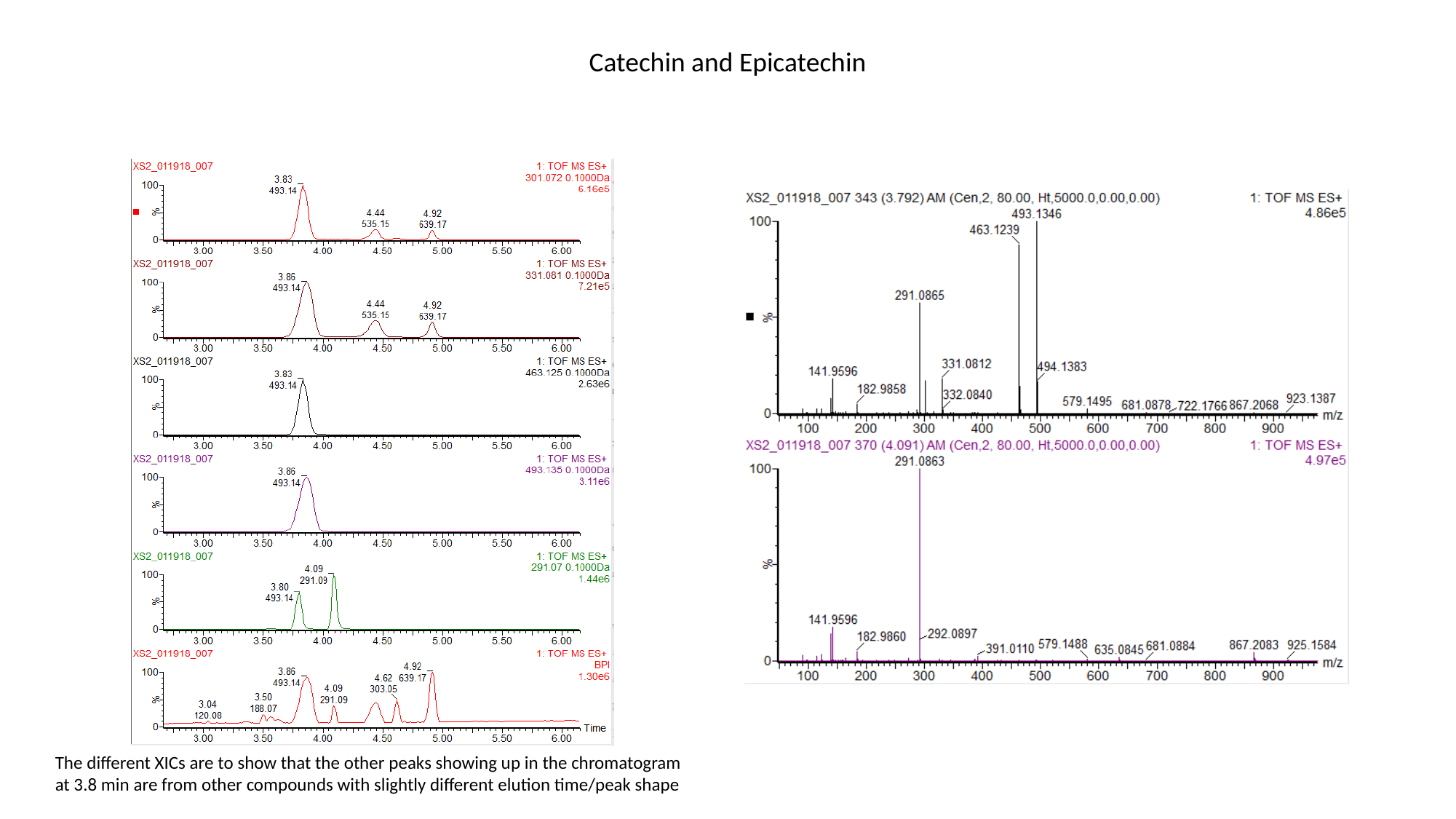

Catechin and Epicatechin
The different XICs are to show that the other peaks showing up in the chromatogram at 3.8 min are from other compounds with slightly different elution time/peak shape

## Slide 2
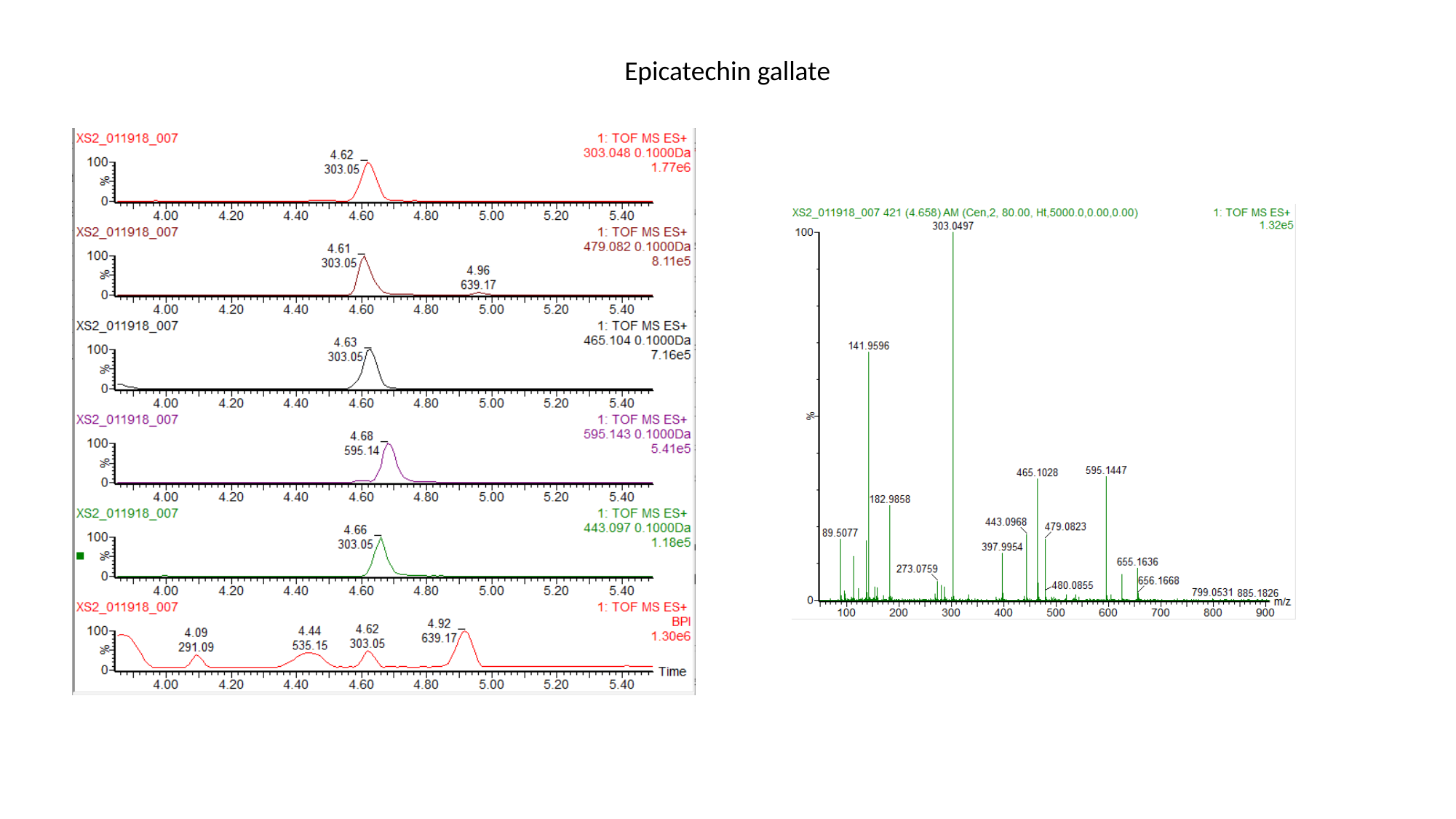

Epicatechin gallate

## Slide 3
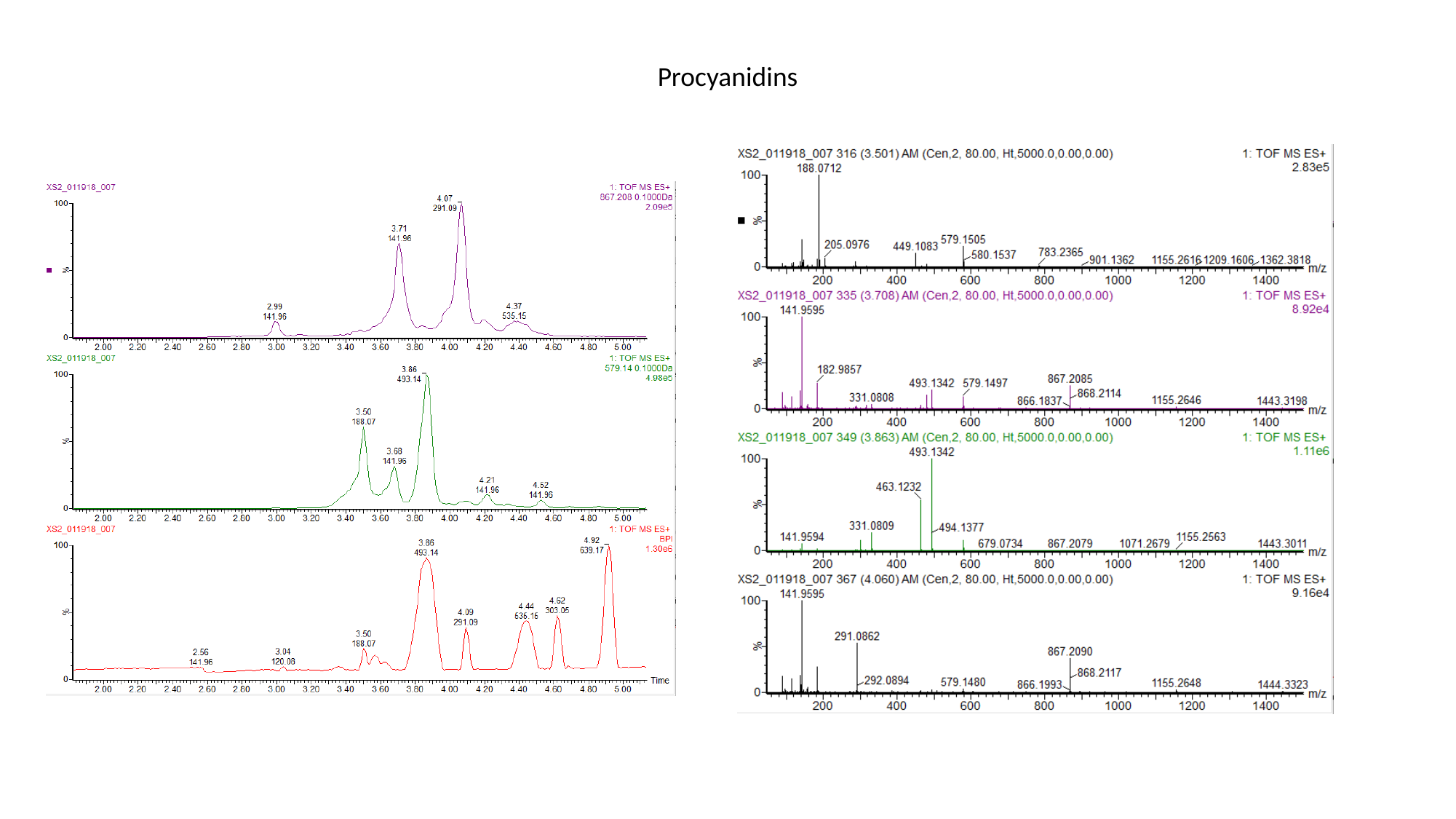

Procyanidins

## Slide 4
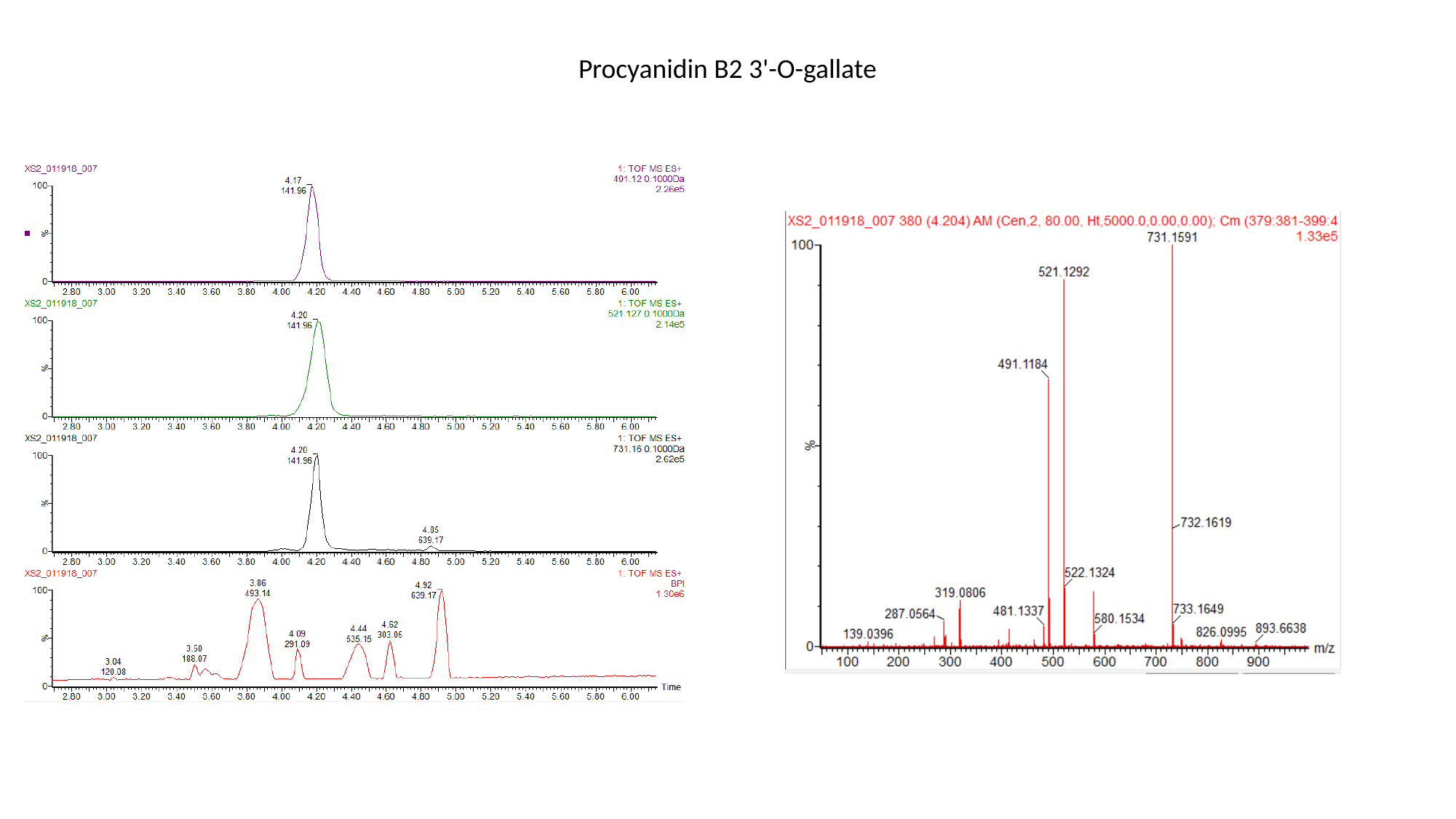

Procyanidin B2 3'-O-gallate
