# Supplementary material for: Influence of freezing and heating conditions on grape seed flavan-3-ol extractability, oxidation, and galloylation pattern
Source: Sci Rep. 2022 Mar 9;12:3838. doi: 10.1038/s41598-022-07925-7 (PMC8907288; doi:10.1038/s41598-022-07925-7)
Supplement: Supplementary file 2 — Supplementary Information. [file 41598_2022_7925_MOESM2_ESM.pdf]

Supplemental Figure 1. Impact of freezing and heating treatments on seed color lightness (L*) development over 24 hours in Pinot noir (A, C) and Cabernet Sauvignon (B, D) seeds at veraison (A, B) and harvest (C, D). Data were analyzed by one-way ANOVA with treatment as the factor, and when differences were significant, means were separated with Tukey's HSD test ($p < 0.05$). Different letters identify significantly different means. $T_{20}I_{20}$, no freezing and thawing at 20 °C; $T_{20}I_{40}$, no freezing and thawing at 40 °C; T-$_{20}$I$_{20}$, freezing at -20 °C and thawing at 20 °C; T-$_{20}$I$_{40}$, freezing at -20 °C and thawing at 40 °C; sampling time; T, freezing temperature; I, incubation temperature. Data is expressed as mean standard ± deviation.

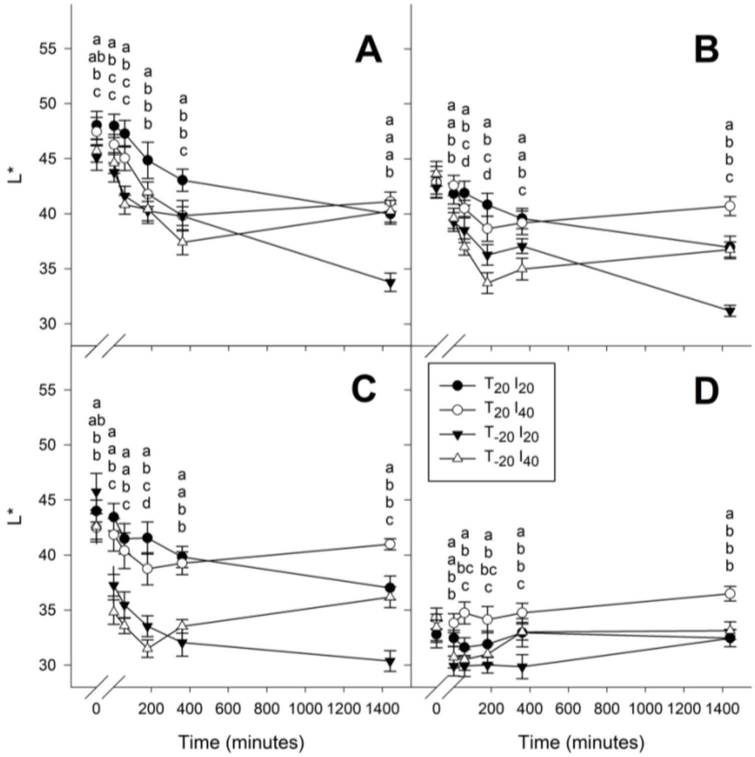

Supplemental Figure 2. Impact of freezing and heating treatments on seed color chroma (C*) development over 24 hours in Pinot noir (A, C) and Cabernet Sauvignon (B, D) seeds at veraison (A, B) and harvest (C, D). Data were analyzed by one-way ANOVA with treatment as the factor, and when differences were significant, means were separated with Tukey's HSD test ($p < 0.05$). Different letters identify significantly different means. $T_{20}I_{20}$, no freezing and thawing at 20 °C; $T_{20}I_{40}$, no freezing and thawing at 40 °C; $T_{-20}I_{20}$, freezing at -20 °C and thawing at 20 °C; $T_{-20}I_{40}$, freezing at -20 °C and thawing at 40 °C; sampling time; T, freezing temperature; I, incubation temperature. Data is expressed as mean standard ± deviation.

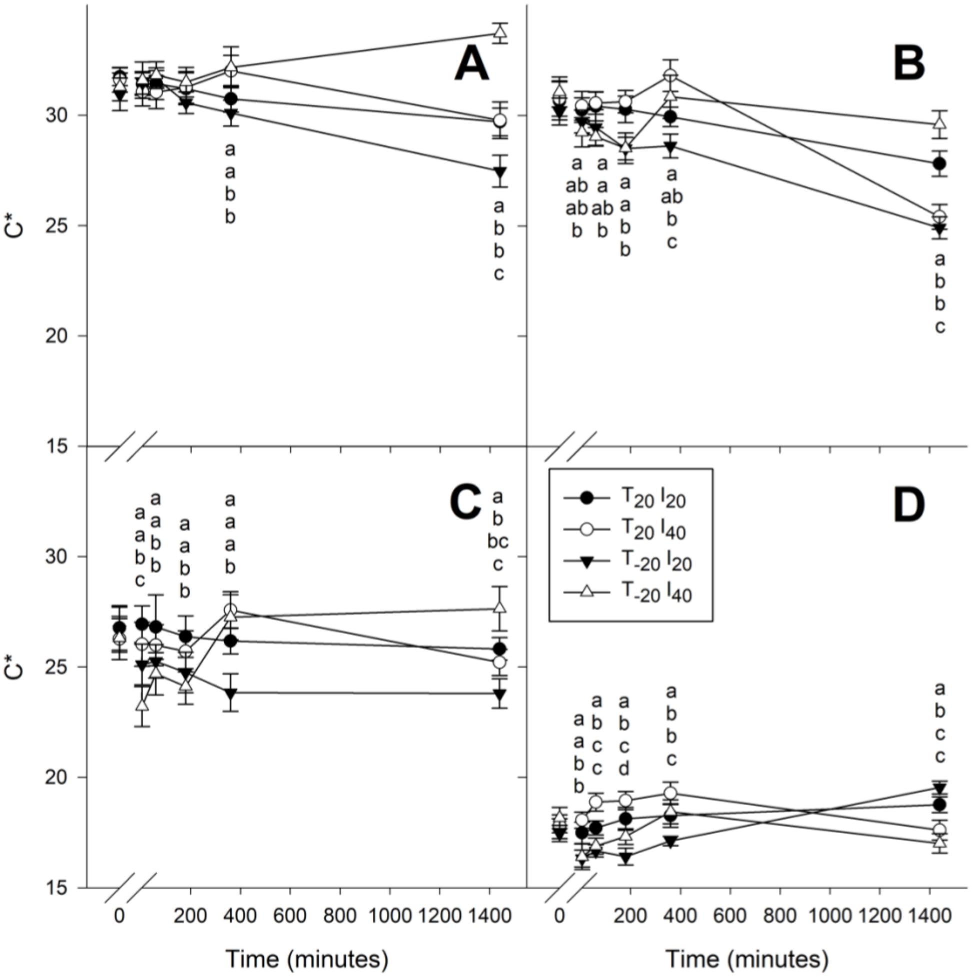

Supplemental Figure 3. Impact of freezing and heating treatments on seed color hue (h*) development over 24 hours in Pinot noir (A, C) and Cabernet Sauvignon (B, D) seeds at veraison (A, B) and harvest (C, D). Data were analyzed by one-way ANOVA with treatment as the factor, and when differences were significant, means were separated with Tukey's HSD test ($p < 0.05$). Different letters identify significantly different means. $T_{20}I_{20}$, no freezing and thawing at 20 °C; $T_{20}I_{40}$, no freezing and thawing at 40 °C; $T_{-20}I_{20}$, freezing at -20 °C and thawing at 20 °C; $T_{-20}I_{40}$, freezing at -20 °C and thawing at 40 °C; sampling time; T, freezing temperature; I, incubation temperature. Data is expressed as mean standard ± deviation.

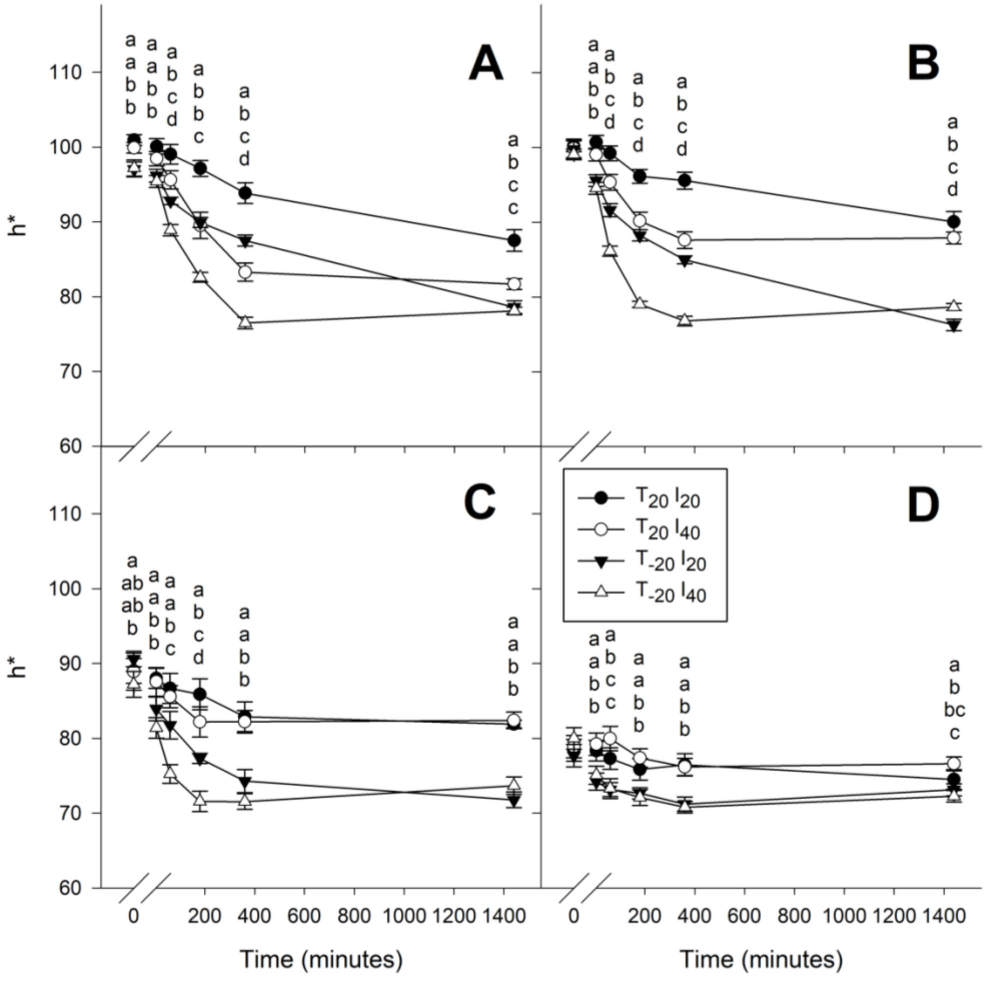

Supplemental Figure 4. Impact of freezing and heating treatments on seed color development (index generated from L*, C*, h* data) over 24 hours in A) Pinot noir (A, C) and Cabernet Sauvignon (B, D) seeds at veraison (A, B) and harvest (C, D). Data were analyzed by one-way ANOVA with time as the factor, and when differences were significant, means were separated with Tukey's HSD test (p < 0.05). Different letters identify significantly different means. $T_{20}I_{20}$, no freezing and thawing at 20 °C; $T_{20}I_{40}$, no freezing and thawing at 40 °C; $T_{-20}I_{20}$, freezing at -20 °C and thawing at 20 °C; $T_{-20}I_{40}$, freezing at -20 °C and thawing at 40 °C; sampling time; T, freezing temperature; I, incubation temperature. Data is expressed as mean standard ± deviation.

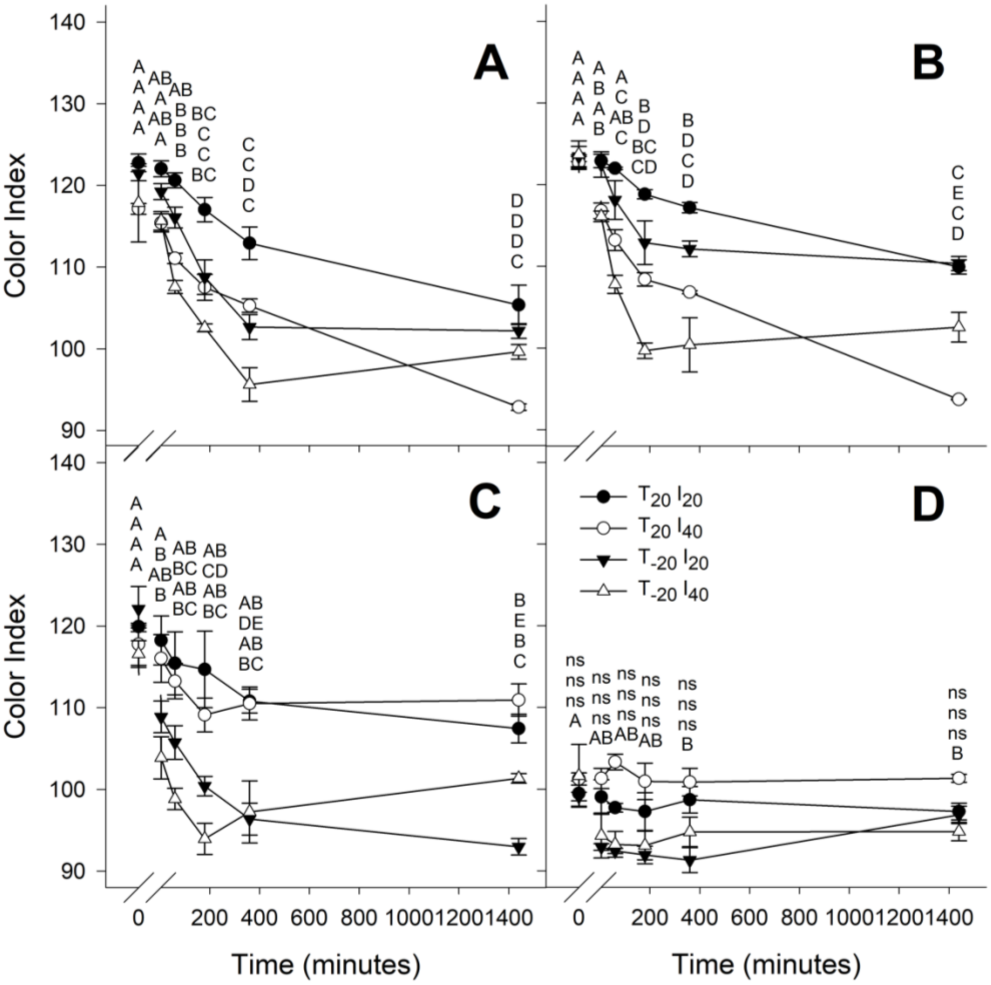

Supplementary Figure 5. Correlation between seed water content (%) and A) the color index and B) flavan-3-ol concentrations of extracts of Pinot noir and Cabernet Sauvignon seeds. All data was utilized for these correlations.

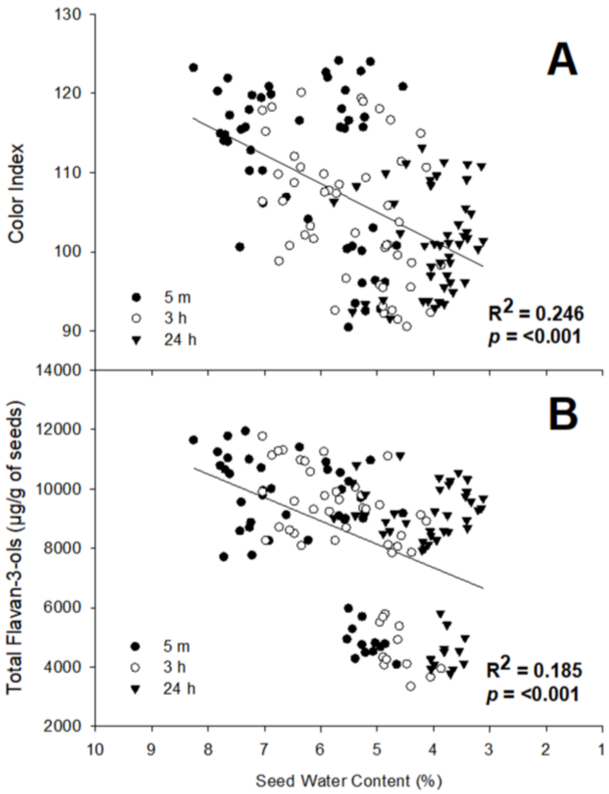

Supplementary Figure 6. Correlation between the change in total phenolics from the control treatment with the percentage of lost water content in seeds of A) Pinot noir at veraison, B) Cabernet Sauvignon at veraison, C) Pinot noir at harvest, and D) Cabernet Sauvignon at harvest. Data from veraison and harvest was combined.

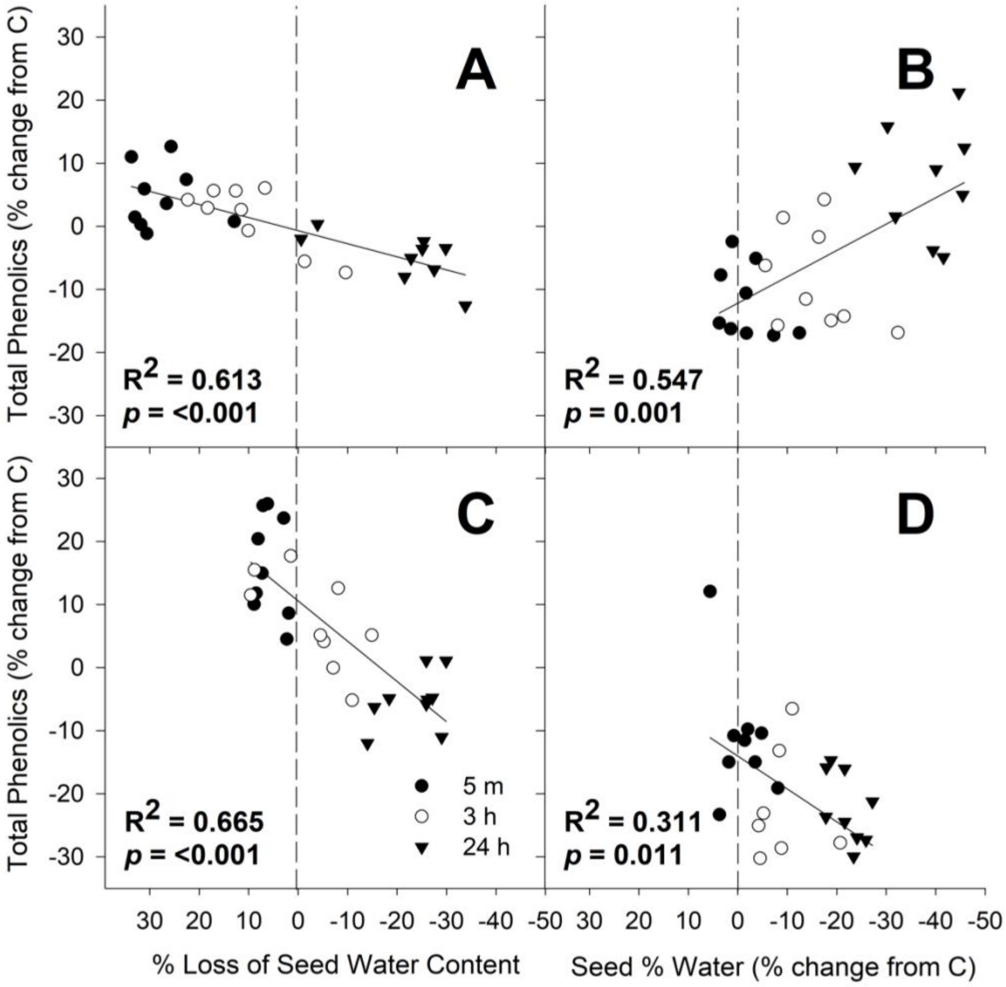

Supplemental Table 1. Basic fruit quality analysis of Pinot noir and Cabernet Sauvignon grapes at veraison and harvest.

	Veraison			Harvest		
**Cultivar[a]**	**TSS (°Brix)**	**pH**	**Total Acidity (g/L)**	**TSS (°Brix)**	**pH**	**Titratable Acidity (g/L)**
Pinot noir	11.6	2.70	18.4	17.0	3.11	9.43
Cabernet Sauvignon	11.1	2.66	16.3	18.5	3.10	5.08

Supplementary Table 2. Influence of natural seed ripening on flavan-3-ols concentration (µg/g seeds) in Pinot noir and Cabernet Sauvignon seed extracts.

**Cultivar**	**Pinot noir**			**Cabernet Sauvignon**		
**Compound**	**Veraison**	**Harvest**	**% change**	**Veraison**	**Harvest**	**% change**
Total Phenolics	10775	8411	-21.9%***[1]	9647	5460	-43.4%***
Flavan-3-ols						
(+)- catechin	3379	2441	-27.8%***	2674	1761	-34.1%***
(-)- epicatechin	4140	3354	-19.0%***	3226	2149	-33.4%***
epicatechin gallate	1836	1312	-28.5%***	2432	582	-76.1%***
total flavan-3-ols	9355	7107	-24.0%***	8332	4492	-46.1%***
Procyanidins						
procyanidin A	21.0	21.1	0.476%	9.59	12.1	26.2%
procyanidin B	729	663	-9.05%	563	569	1.07%
procyanidin B2 3'-*O*-gallate	257	223	-13.2%*	404	129	-68.1%***
procyanidin C	334	326	-2.40%	230	197	-14.3%**
procyanidin C2 gallate	78.9	70.4	-10.8%	108	60.8	-43.7%***
total procyanidins	1420	1304	-8.17%	1315	968	-26.4%***

[1]Treatments changed significantly from the control ($T_{20}I_{20}$) are marked with an asterisk, *, $p<0.05$; **, $p<0.01$; ***, $p<0.001$.

Supplementary Table 3. Influence of seed freezing (-20 ºC) on flavan-3-ols concentration (µg/g seeds) in Pinot noir and Cabernet Sauvignon seed extracts.

**Cultivar**	**Pinot noir**			**Cabernet Sauvignon**		
**Compound**	**20 ºC**	**-20 ºC**	**% change**	**20 ºC**	**-20 ºC**	**% change**
Total Phenolics	9705	9701	-0.04%	7253	6654	-8.26%
Flavan-3-ols						
(+)- catechin	2930	3049	+4.05%	2132	2053	-3.72%
(-)- epicatechin	3766	3950	+4.87%	2569	2531	-1.50%
epicatechin gallate	1612	1289	-20.1%***[1]	1442	1009	-30.0%*
total flavan-3-ols	8308	8288	-0.241%	6143	5593	-8.95%
Procyanidins						
procyanidin A	18.7	13.1	-29.9%***	9.36	6.40	-31.6%***
procyanidin B	714	740	+3.59%	547	541	-1.16%
procyanidin B2 3'-*O*-gallate	248	239	-3.43%	262	236	-9.73%
procyanidin C	342	346	+1.11%	211	211	-0.05%
procyanidin C2 gallate	74.4	74.6	+0.265%	81.0	67.0	-17.2%**
total procyanidins	1397	1413	+1.15%	1110	1061	-4.41%

[1]Treatments changed significantly from the control ($T_{20}I_{20}$) are marked with an asterisk, *, $p<0.05$; **, $p<0.01$; ***, $p<0.001$.

Supplementary Table 4. Influence of seed heating (40 ºC) during thawing on flavan-3-ols concentration (µg/g seeds) in Pinot noir and Cabernet Sauvignon seed extracts.

**Cultivar**	**Pinot noir**			**Cabernet Sauvignon**		
**Compound**	**20 ºC**	**40 ºC**	**% change**	**20 ºC**	**40 ºC**	**% change**
Total Phenolics	9724	9680	-0.452%	7133	6774	-5.03%
Flavan-3-ols						
(+)- catechin	3010	2969	-1.37%	2131	2055	-3.57%
(-)- epicatechin	3855	3861	+0.175%	2616	2484	-5.03%
epicatechin gallate	1473	1427	-3.11%	1270	1180	-7.09%
total flavan-3-ols	8338	8257	-0.971%	6017	5719	-4.95%
Procyanidins						
procyanidin A	17.9	13.9	-22.2%*[1]	8.76	7.00	-20.1%
procyanidin B	716	738	+3.00%**	561	527	-6.10%**
procyanidin B2 3'-*O*-gallate	241	245	+1.67%	255	243	-4.86%
procyanidin C	337	351	+4.03%*	214	207	-3.04%***
procyanidin C2 gallate	73.8	75.2	+1.88%	76.9	71.1	-7.55%
total procyanidins	1386	1423	+2.67%**	1116	1055	-5.47%*

[1]Treatments changed significantly from the control ($T_{20}I_{20}$) are marked with an asterisk, *, $p<0.05$; **, $p<0.01$; ***, $p<0.001$.
